# SELF-REPORTED DENTAL CONDITIONS AND MEMORY DECLINE: THE MEDIATION ROLE OF STROKE

**DOI:** 10.1093/geroni/igac059.1098

**Published:** 2022-12-20

**Authors:** Chenxin Tan, Huabin Luo, Xiang Qi, Bei Wu

**Affiliations:** New York University, New York, New York, United States; East Carolina University, Greenville, North Carolina, United States; New York University, New York City, New York, United States; New York University, New York, New York, United States

## Abstract

The relationship between oral health and cognitive function has been studied extensively; however, little research has examined the underlying pathways. Using a cohort of 6,403 adults aged 51+ from the 2006-2010 English Longitudinal Study of Ageing, we analyzed the effect of changes in self-rated dental conditions on memory function (ranges 0-20) and the mediation effect of stroke using a causal mediation analysis framework. Controlling for socio-demographics, lifestyle, and health status, we found that compared with participants whose dental conditions remained the same, those who reported deterioration of dental conditions in 2006-2010 (N=1,755) experienced a steeper decline in memory function, with an average total effect of -0.22 (95% CI=-0.36, -0.07). The stroke incident had an average mediation effect of -0.007 (95% CI=-0.014, -0.001), representing 3% of the average total effect. The findings accentuate the importance of dental care access for older adults with exacerbated oral health problems.

